# Quantifying Soil Complexity Using Fisher Shannon Method on 3D X-ray Computed Tomography Scans

**DOI:** 10.3390/e25101465

**Published:** 2023-10-19

**Authors:** Domingos Aguiar, Rômulo Simões Cezar Menezes, Antonio Celso Dantas Antonino, Tatijana Stosic, Ana M. Tarquis, Borko Stosic

**Affiliations:** 1 Departamento de Estatística e Informática, Universidade Federal Rural de Pernambuco, Rua Dom Manoel de Medeiros s/n, Dois Irmãos, Recife 52171-900, PE, Brazil; 2 Departamento de Energia Nuclear, Universidade Federal de Pernambuco, Av. Prof. Moraes Rego 1235, Cidade Universitária, Recife 50670-901, PE, Brazil; 3 Department of Applied Mathematics, ETSIAAB, Universidad Politécnica de Madrid, Av. Puerta de Hierro, n. 2-4, 28040 Madrid, Spain; anamaria.tarquis@upm.es; 4 CEIGRAM, ETSIAAB, Universidad Politécnica de Madrid, Av. Puerta de Hierro, n. 2-4, 28040 Madrid, Spain

**Keywords:** complexity, Fisher Shannon plane, land use change, X-ray CT scan soil samples

## Abstract

The conversion of native forest into agricultural land, which is common in many parts of the world, poses important questions regarding soil degradation, demanding further efforts to better understand the effect of land use change on soil functions. With the advent of 3D computed tomography techniques and computing power, new methods are becoming available to address this question. In this direction, in the current work we implement a modification of the Fisher–Shannon method, borrowed from information theory, to quantify the complexity of twelve 3D CT soil samples from a sugarcane plantation and twelve samples from a nearby native Atlantic forest in northeastern Brazil. The distinction found between the samples from the sugar plantation and the Atlantic forest site is quite pronounced. The results at the level of 91.7% accuracy were obtained considering the complexity in the Fisher–Shannon plane. Atlantic forest samples are found to be generally more complex than those from the sugar plantation.

## 1. Introduction

The degradation of soils due to land use changes driven by economic factors represents a major concern for the foreseeable future in many parts of the world. More precisely, land use change may adversely affect fundamental soil functions, such as nutrient storage, diffusion and cycling, carbon storage and greenhouse gas emissions, erosion resistance, water storage, drainage, and filtration [1,2,3,4,5]. Moreover, the biodiversity of forests may also be unfavorably affected by systematic land use change [6]. On the other hand, poverty and population growth lead to an ever-increasing demand for indiscriminate natural resources in developing countries. The demand for pasture, timber, firewood, and crops drives the conversion of tropical forests into agricultural land at an alarming rate. This situation dictates comprehensive studies on the impact of deforestation and land use conversion on soil quality in general. More precisely, the outstanding question is whether the cultivation of deforested land may lead to the permanent degradation of land productivity. The ecologically sensitive components of the tropical ecosystem may not buffer the effects of agricultural practices (see, e.g., [7] and references therein). Therefore, comprehensive assessment of soil properties is fundamental for the early detection and mitigation of adverse soil change effects.

The effects of land use change have been addressed mainly focusing on physical, chemical, and biological properties [7,8,9], while far fewer studies have been devoted to changes in soil structure [10,11]. The latter governs its functions [12], and quantification of soil architecture can be seen as a key to better understanding the complex dynamical phenomena that govern these functions. Therefore, a comprehensive description and quantification of soil functions rely on an in-depth understanding of characteristics such as the three-dimensional distribution of constituents, connectedness, hierarchical organization, and complexity.

While X-ray computed tomography (CT) has been advancing at an impressive rate over the last decades, it has also become a widespread tool for non-destructive 3D soil visualization and quantification, shedding new light on soil functions [13]. The diverse properties of soil that have not been previously amenable to analyses can now be assessed through CT scans, providing novel fundamental insights into soil functions [13]. These properties include isotropy, homogeneity, complexity, and the hierarchical fractal (or multifractal) organization of soil constituents, contributing to a deeper understanding of soil’s physical, chemical, and biological processes [14]. X-ray CT scans have already been studied to characterize the pores’ spatial distribution, revealing extraordinary complexity of the pore space [15,16,17,18]. The complexity of soil structure has also been addressed through methods based on concepts from statistical physics and information theory, such as fractals and multifractals [19,20,21], information content [22] and complex networks [23,24]. As a consensus has not yet been reached on the adequate threshold for separating pores from solid in CT scans [25], it has also been suggested that rather than thresholding, grayscale soil images should be used for the multifractal characterization of the soil structure [26,27,28,29,30].

Between 2000 and 2018, Brazil suffered a total reduction of 489,877 km^2^ in the natural area of its six terrestrial biomes. Among them, the Atlantic Forest biome has the one with the highest percentage of degradation over time, as it covers the most industrialized and productive areas, in addition to having the highest demographic density in the national territory, housing about 49.3% of the urban areas of the country [31]. One of the crops that most stands out in the region of the Atlantic Forest biome is sugarcane, especially in the northeast region of the country, where cultivation is present in eight of the nine states in the region. For the 2020/2021 harvest, an increase of 1.6% in the planted area and 4.1% in production were estimated compared to the previous sugarcane harvest in the northeastern region of Brazil [32]. The replacement of the native vegetation of the Atlantic Forest with sugarcane cultivation generates negative impacts on the physical attributes of the soil [33,34,35,36]. These attributes control many soil functions, such as water retention and infiltration, gas exchange, resistance to erosion, nutrient dynamics, and root penetration [12], and directly influence ecosystem services.

In this work, we investigate how land use change affects soil structure by using information theory to quantify the complexity of soil 3D X-ray CT soil samples. For the first time, the Fisher–Shannon method [37], introduced to jointly quantify the local and global properties of the probability density function of unidimensional signals, is applied in the context of soil complexity. In the current study, the “signals” are represented by a 790 × 790 set of 1d vertical lines of 790 greyscale values in X-ray CT scan images of soil samples from a sugarcane field, and a nearby Atlantic forest site, in northeastern Brazil. For each image and each of these sequences, we calculate Shannon entropy power (SEP) and Fisher information measure (FIM) that quantify the disorder and structural organization of a signal’s variation [38]. The joint FIM/SEP analysis is then performed through the Fisher–Shannon information plane (FS) via an innovative normalization procedure to achieve a 91.7% level of accuracy of distinction between the sugar plantation and the Atlantic forest samples.

## 2. Methodology

### 2.1. Soil Samples

**Figure 1:**
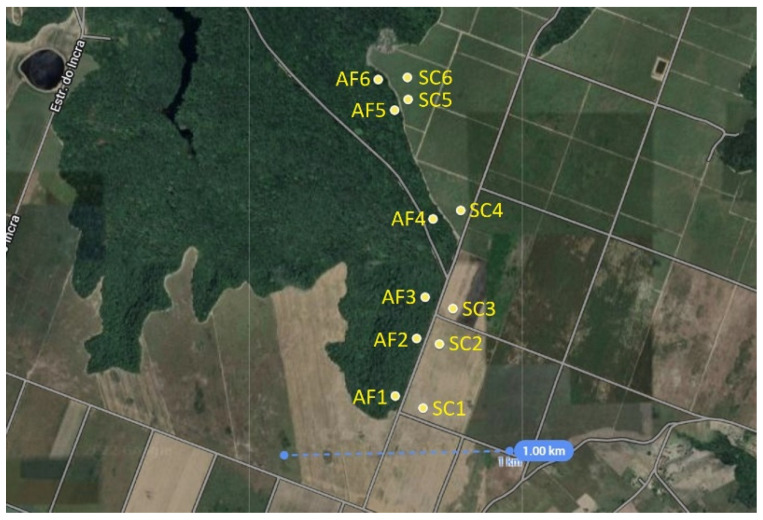
Spatial distribution of sample sites. Two samples were taken at each site: one at 10 and another at 20 cm depth.

Twenty-four soil samples analyzed in this work were collected from sugarcane cultivation and a nearby native Atlantic forest at a location in the northeastern Brazilian region, the state of Pernambuco, between latitudes −7.84836 and −7.83519 and longitudes −34.9973 and −34.9935, as shown in Figure 1. Two samples were collected at each site: one at 10 cm and another at 20 cm depth.

The samples were collected using a soil auger with an internal PVC cylinder of 7.5 cm height and 7.5 cm diameter and excavated by careful penetration with a cylinder coupled with a blade. After the insertion of the auger in the soil, the cylinders were carefully extracted to ensure the preservation of the original structure of the environment inside the PVC cylinders. The samples were then dried at 40 °C to remove the water content before the scanning tomography of the samples.

The CT tomography was performed using a third-generation Nikon XT H 225 ST X-ray microtomograph with 150 kV voltage, 180 μA current, 500 ms exposure time, and a 45 μm resolution for voxels. A copper filter with a thickness of 0.5 mm was used to minimize low-intensity photons. After scanning the total cylinder volume in the initial acquisition, a subvolume of interest was defined and reconstructed using CTPro 3D XT 3.0.3 (Nikon Metrology NV, Brighton, MI, USA) software. The central part of the cylinder was highlighted to avoid edge influence. The reconstructed 2D axial projections maintained the spatial resolution of the acquisition of 45 μm and were saved at a radiometric resolution (grayscale level) of 16 bits. The final volume was 790 stacks with 790 × 790 pixels, with an end volume of 790^3^ = 493,039,000 voxels.

The voxel values of the CT scan images correspond to local sample density on Hounsfield unit (HU) scale (a linear transformation of the linear attenuation coefficient measurement such that the radiodensity of water is defined as 0 HU, and the radiodensity of air is defined as −1000 HU). The minimum observed HU value is 0, the maximum is 32,492, mean is 16,327, and the standard deviation is 402.

Considering the vertical (gravity) direction as naturally preferential from a phenomenological point of view, in what follows, we perform calculations on 790 × 790 = 624,100 vertical lines of 790 grey-level values each for every sample.

### 2.2. The Fisher–Shannon Method

The Fisher–Shannon method consists of a joint analysis of Fisher information measure (FIM), which quantifies the amount of organization (or order) in a signal, and Shannon entropy (SE) which quantifies the amount of disorder [37]. Fisher introduced the FIM concept in the statistical estimation theory [39], and it was subsequently used to describe physical systems [40,41], as well as for time series analysis in geophysics [42,43], ecology [44], astrophysics [45], meteorology [46,47], hydrology [48], and social science [49].

For a univariate distribution of a continuous variable X with probability density function (PDF) f(x), the Fisher information measure $I_{X}$ is defined as [49]

(1) $$I_{x}=\int_{-\infty }^{\infty }{\left(\frac{\partial }{\partial x}f(x)\right)}^{2}\frac{1}{f(x)}dx\;,\;$$

and Shannon entropy $H_{X}$ as

(2) $$H_{x}=-\int_{-\infty }^{\infty }f\left(x\right)\log f\left(x\right)dx\;.\;$$

The Fisher information measure thus describes the local properties of the PDF, while the Shannon entropy describes its global properties [49]. The shape of the PDF is reflected on these measures, as the FIM assumes high values if the PDF is narrow and low values if the PDF is broad, while SE attains high values for a broad PDF and low values for a narrow PDF.

Instead of Shannon entropy, it is often more convenient [50] to use the quantity called Shannon entropy power (SEP) defined by

(3) $$N_{x}=\frac{1}{2\pi e}e^{2H_{x}}\;.\;$$

The product $C_{X}=N_{X}I_{X}$ satisfies “isoperimetric inequality” $N_{X}I_{X}\geq 1$ (where equality holds for the Normal distribution), demonstrating that FIM and Shannon entropy are intrinsically related and can be jointly used to characterize the non-stationary behavior of complex signals. The product $N_{X}I_{X}$ is called Fisher–Shannon complexity (FSC) and can be used as a statistical measure of the complexity of the signal under study [51]. The joint FIM/SEP analysis is performed through the Fisher–Shannon information plane (FS), where Shannon entropy power $N_{X}$ is used for the horizontal axes, and Fisher information measure $I_{X}$ is taken for the vertical axis variable [37]. The signal is mapped to the point with coordinates ${(N}_{X},I_{X})$, which can lie anywhere in the FS plane where the “isoperimetric inequality” $N_{X}I_{X}\geq 1$ is satisfied. The distance from the “isocomplexity” line $N_{X}I_{X}=1$ can be used as a measure of the complexity of the signal [49].

As the above measures depend only on the PDF, Fisher–Shannon analysis can be implemented for real-world datasets corresponding to complex systems through nonparametric density estimation, avoiding parametric assumptions on the distribution. One possibility is using histograms with the discretized version of (1) and (2). However, in this work, we implement kernel density estimation, which is more reliable [52] in the current case. The Kernel density estimator of the PDF is given by [53]

(4) $${\hat{f}}_{M}\left(x\right)=\frac{1}{Nb}\sum_{i=1}^{N}K\left(\frac{x-x_{i}}{b}\right)\;,\;$$

where b>0 is the so-called bandwidth parameter, N is the length of the signal, and K(u) is the kernel function, which is a continuous symmetric function that satisfies K(u)≥0 and $\int_{-\infty }^{+\infty }K\left(u\right)du=1$. The most widely used is the Gaussian kernel $K\left(u\right)={\left(2\pi \right)}^{-1/2}exp\left(-u^{2}/2\right)$ yielding

(5) $${\hat{f}}_{M}\left(x\right)=\frac{1}{Nb\sqrt{2\pi }}\sum_{i=1}^{N}e^{-\frac{{\left(x-x_{i}\right)}^{2}}{2b^{2}}}\;.\;$$

The term “nonparametric density estimation” here refers to avoiding a choice of the functional form of the distribution and the corresponding parameter estimation. On the other hand the bandwidth parameter b>0 is used here to control the smoothness of the PDF and is determined here through Silverman’s rule [54]

(6) $$b=0.9\underset{}{\min}\left(\sigma ,\frac{IQR}{1.34}\right)n^{-\frac{1}{5}}\;,\;$$

where n is the sample size, σ is standard deviation, and *IQR* is the interquartile range.

## 3. Results and Discussion

The $I_{x}$, $N_{x}$, and $C_{x}$ values were calculated for each of the 624,100 vertical lines of length 790 for all the 24 CT scan images, as well as for the 790 horizontal planes of 790 × 790 = 624,100 voxels each, and for the full 790^3^ = 493,039,000 voxel images. The PDF f(x) in the 1d case corresponds to 790 voxels for each of the 624,100 vertical lines; in the 2d case, f(x) corresponds to 624,100 voxels for each of the 790 vertical planes; and in the 3D case, there is a single PDF f(x) corresponding to all the 493,039,000 voxels.

**Figure 2:**
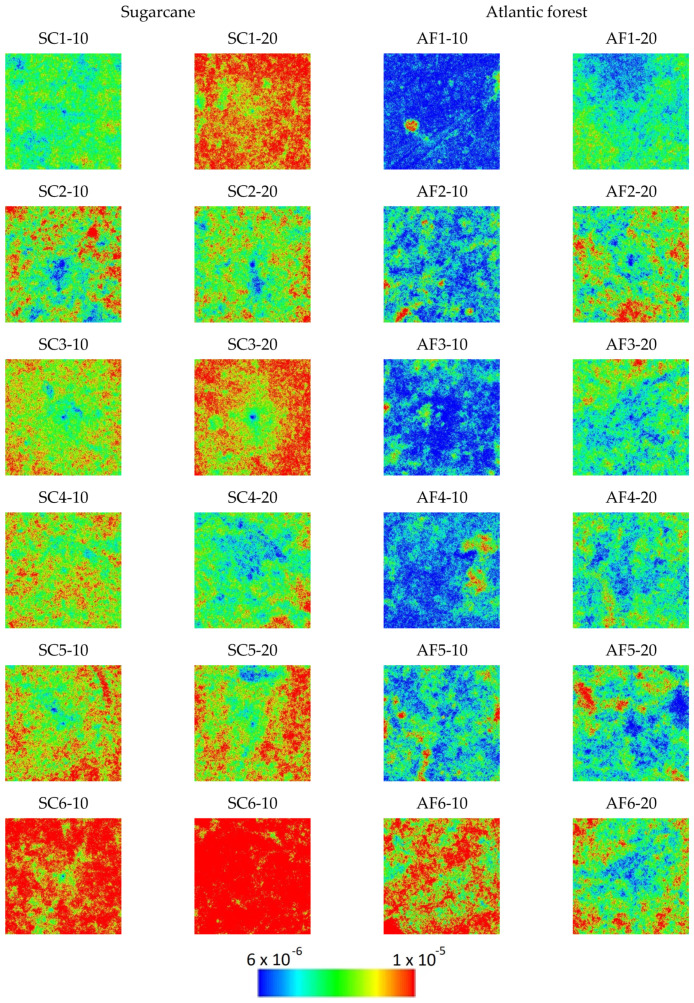
Spatial distribution of the $I_{x}$ values in the horizontal projection plane for each sample. Pixels are color-coded in blue for values below two standard deviations from the global average and red for values above two standard deviations. For values in between, a spectrum of colors is used, as per the color bar.

**Figure 3:**
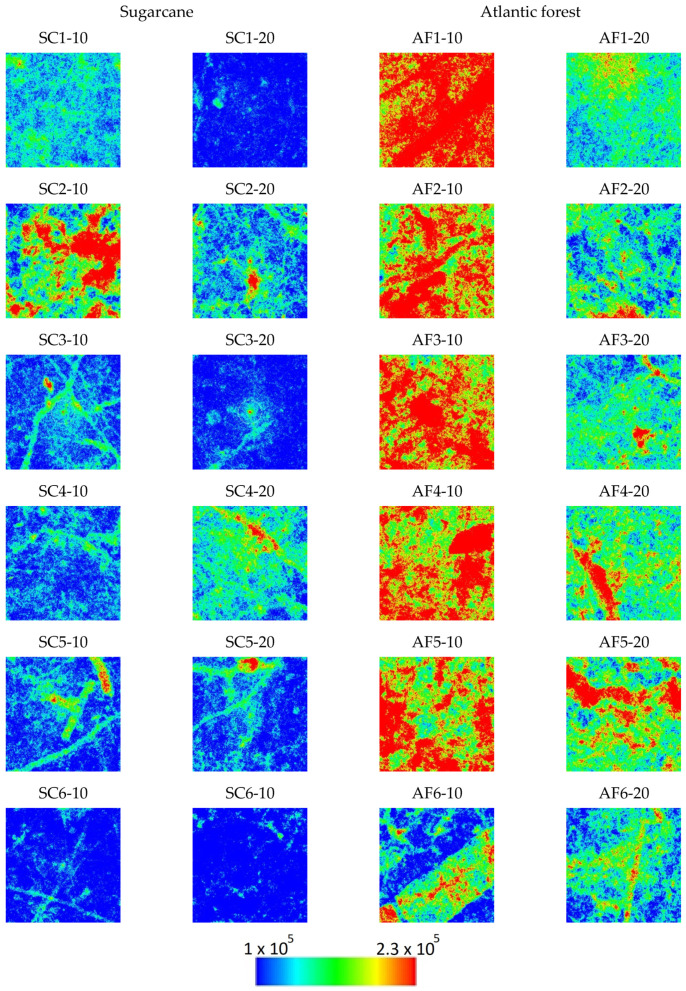
Spatial distribution of the $N_{x}$ values in the horizontal projection plane for each sample. Pixels are color-coded in blue for values below two standard deviations from the global average and red for values above two standard deviations. For values in between, a spectrum of colors is used, as per the color bar.

**Figure 4:**
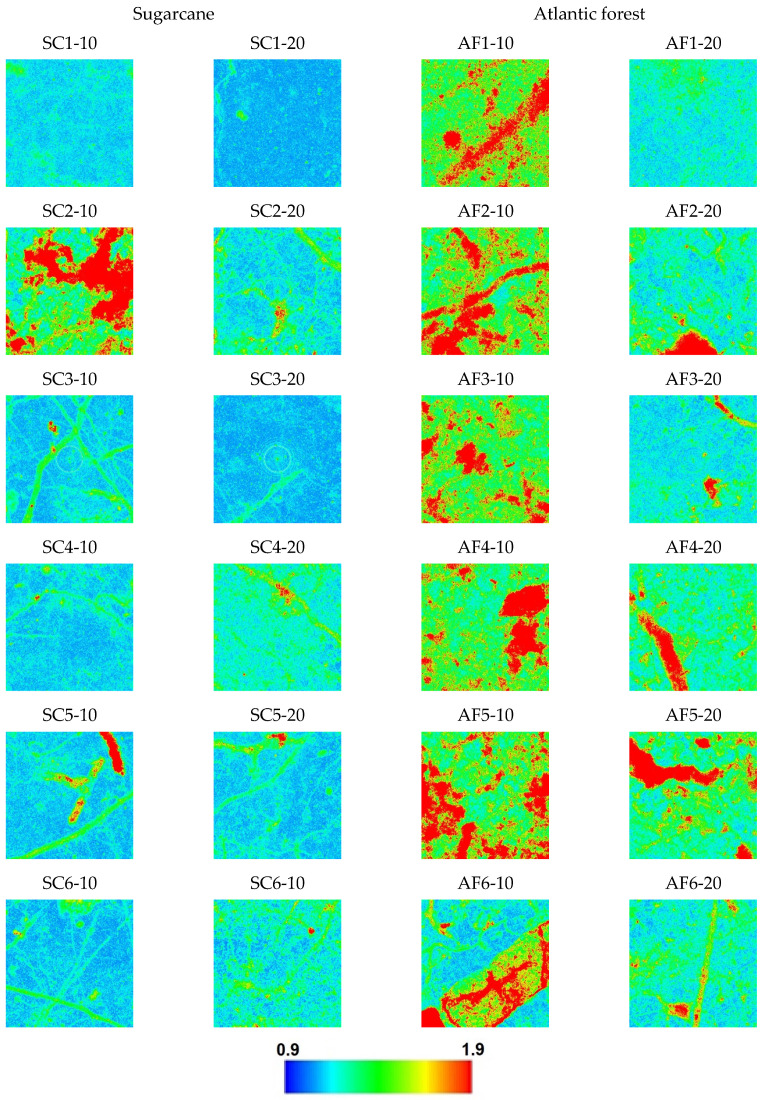
Spatial distribution of the $C_{x}$ values in the horizontal projection plane for each sample. Pixels are color-coded in blue for values below two standard deviations from the global average and red for values above two standard deviations. For values in between, a spectrum of colors is used, as per the color bar.

After extensive testing with different combinations of quantities and distribution measures that can be extracted from these calculations, we have found that the first option of considering the set of vertical lines for each sample yields the best distinction between the sugar cane and the Atlantic forest samples. The descriptive statistics (minimum, maximum, quartiles, mean, and standard deviation) of the $I_{x}$, $N_{x}$, and $C_{x}$ values of vertical lines, obtained with bandwidth b=94.85 (average Silverman’s rule value for all the vertical strips of all the images), are presented in Table A1, Table A2 and Table A3 in the Appendix A, respectively, and the distribution of the values in the 790 × 790 plane for all samples are presented in Figure 2, Figure 3 and Figure 4.

As seen in Figure 2, Figure 3 and Figure 4, the spatial distribution of $I_{x}$ demonstrates higher values for sugarcane (SC) samples compared to the Atlantic forest (AF), while the $N_{x}$ distribution demonstrates the opposite, i.e., lower values are found for SC in comparison with the AF samples. On the other hand, these differences are mostly canceled out when the complexity $C_{X}=N_{X}I_{X}$ is calculated; the SC samples $C_{X}$ values are found to be rather homogeneous except for the sample SC2-10, while inhomogeneity is rather pronounced for the AF samples, more so for the 10 cm than for the 20 cm depth samples. These findings are purely phenomenological, and the observations may not hold in a general scenario of native versus cultivated land samples.

**Figure 5:**
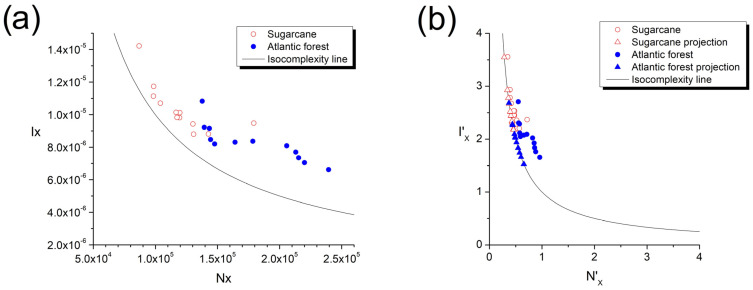
(**a**) Scatterplot of the original $\left(N_{X}{,\;I}_{X}\right)$ value pairs, and (**b**) scatterplot of the normalized values $\left(N_{X}^{\prime },I_{X}^{\prime }\right)$, together with projections $\left(N_{X0}^{\prime },I_{X0}^{\prime }\right)$ to the closest point on the isocomplexity line.

In order to address the distance from the isocomplexity line $N_{X}I_{X}=1$ as a measure of complexity of the vertical sample lines [49], it should be noted that the scales of Shannon entropy power with an average of $\langle N_{x}\rangle =1.527\times 10^{5}$ and Fisher information with an average of $\langle I\rangle =9.278\times 10^{-6}$ differ in orders of magnitude, while the average of the Fisher–Shannon complexity $\langle C_{x}\rangle =1.352$ is of the order of unity. If the projection of a point $\left(N_{X}{,\;I}_{X}\right)$ in the FS plane presented in Figure 5a to the nearest point on the “isocomplexity” line is denoted by $\left(N_{X0}{,\;I}_{X0}\right)$, then the displacement of Shannon information ${\Delta I}_{X}\equiv I_{X}-I_{X0}$ turns negligible in comparison to the displacement of Shannon entropy power ${\Delta N}_{X}\equiv N_{X}-N_{X0}$, and by minimizing the distance to the isocomplexity line, the points are projected vertically down to the isocomplexity line, with the Euclidean distance being practically reduced to ${\Delta N}_{X}$ and there being no influence of ${\Delta I}_{X}$.

**Figure 6:**
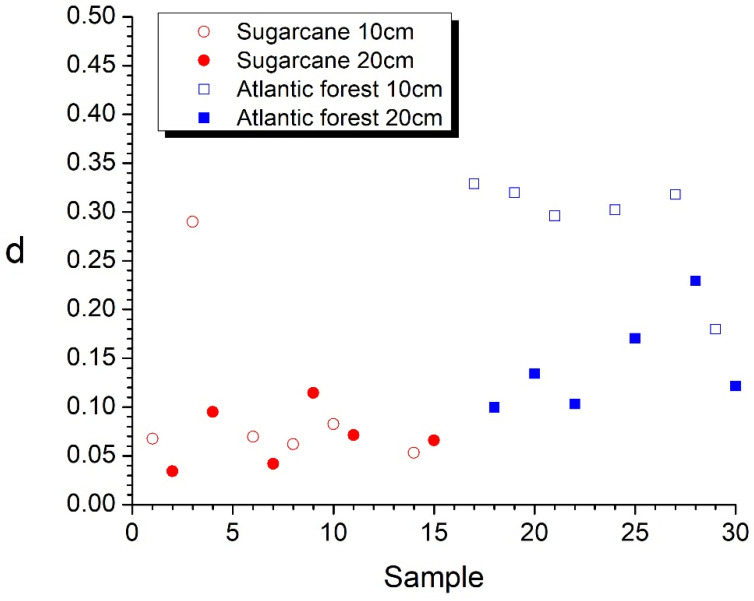
The distances’ scatterplot from the isocomplexity line in the order of samples from south to north (same as in Table 1).

To mitigate this fact, here we introduce a novel normalization procedure for the variables $N_{X}$ and $I_{X}$. More precisely, first we identify the maximum value $N_{Xmax}$ among all the samples, and we scale all the sample values as $N_{X}^{\prime }=N_{X}/N_{Xmax}$ and $I_{X}^{\prime }=I_{X}N_{Xmax}$, thus ***preserving*** the $C_{x}{\equiv N}_{X}I_{X}$ complexity values. Distance from a point $\left(N_{X}^{\prime },I_{X}^{\prime }\right)$ to a projection point $\left(N_{XI}^{\prime },I_{XI}^{\prime }\right)$ on the isocomplexity plane is now given by

(7) $$d=\sqrt{{\left(N_{X}^{\prime }-N_{XI}^{\prime }\right)}^{2}+{\left(I_{X}^{\prime }-\frac{1}{N_{XI}^{\prime }}\right)}^{2}}\;$$

and setting the derivative of d with respect to $N_{XI}^{\prime }$ to zero to find the closest point $\left(N_{X0}^{\prime },I_{X0}^{\prime }\right)$ yields the fourth-order polynomial expression for $x\equiv N_{X0}^{\prime }\equiv 1/I_{X0}^{\prime }$

(8) $$x^{4}-x^{3}N_{X}^{\prime }+{xI}_{X}^{\prime }-1=0\;,\;$$

which is solved numerically for all samples. The results of this novel procedure are presented in Figure 5b, and the distances scatterplot from the isocomplexity plane is presented in Figure 6.

The sugarcane sample’s complexity, quantified by the distance from the isocomplexity line (Figure 4 and Figure 5) is generally lower than those of the Atlantic forest samples, which also exhibit large fluctuations between the alternative values from samples taken at depths of 10 cm and 20 cm. The complexity values are presented in Table 1.

**Figure 7:**
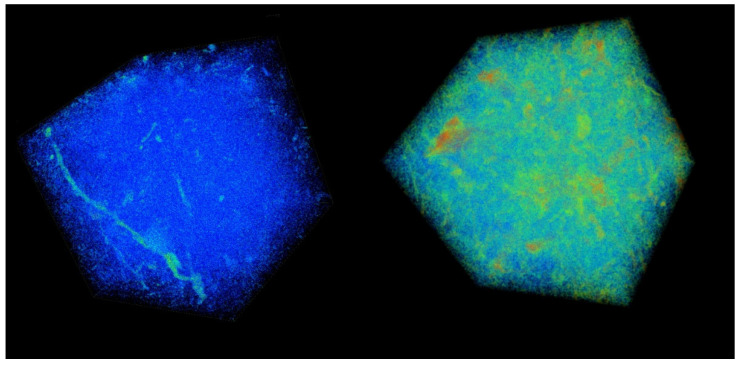
Samples SC1_20 with d = 0.0342 (**left**) and AF1_10 with d = 0.329 (**right**) at a threshold of 1500 Hounsfield units (a common quantitative scale for radiodensity).

To demonstrate the validity of this novel approach, in Figure 7, we show the 3D images of the two samples with the lowest and highest complexity, respectively.

It should be stressed here that the current approach, without using any arbitrary parameters (such as, e.g., threshold), not only yields results that agree well with common sense (as can be seen in Figure 5) but also provides a precise quantitative measure of the complexity of the samples. The usefulness of this approach for quantifying soil degradation should be tested in future studies in the natural environment and controlled laboratory experiments.

**Figure 8:**
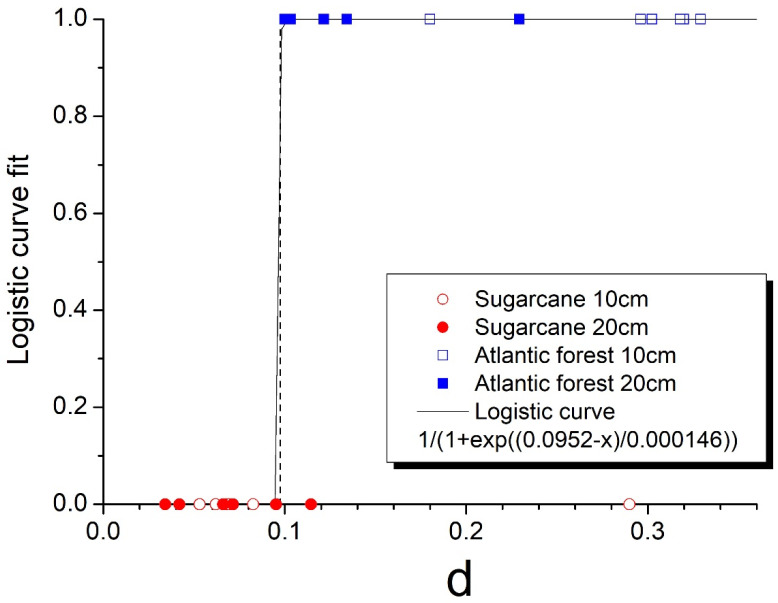
Fit to a logistic function. The vertical dashed line represents the threshold d = 0.0952.

Finally, to test the discriminative power of the current approach, we implement here the fitting of values from Table 1 to a logistic function, where a categorical variable of zero value is attributed to the sugarcane samples and unit value to the Atlantic forest samples, with results shown in Figure 8.

Considering logistic regression as a binary classifier, the threshold of d=0.0952 separates the two groups of samples with only two samples (SC2-10 and SC4-20) falling into the wrong category. It should be noted here that the k-means method does not produce meaningful results in this case because of the difference in the variance of the d values of the two groups, which is much smaller for the sugarcane samples than for the Atlantic forest samples.

Observing the images of $I_{x}$, $N_{x}$, and $C_{x}$ of spatial distribution of these samples in Figure 2, Figure 3 and Figure 4 reveals that the origin of the strikingly high value of d=0.290 for sample SC2-10 stems from the pronounced values of Shannon entropy power $N_{X}$, which, in combination with Information measure values $I_{X}$, yield a Fisher–Shannon complexity $C_{X}$ for each of the 624,100 vertical lines that was rather similar to those of the Atlantic forest samples. Therefore, the correct grouping of 22 out of 24 samples (91.7%) is attained, demonstrating the power of the current novel nonparametric approach. Nevertheless, considering the fact that the samples are geographically very close to each other so that correlation among them could have resulted in overfitting, this obtained accuracy represents the training error, and the regressor is yet to be tested in an independent, uncorrelated dataset.

## 4. Conclusions

Overall, we can claim that the Fisher–Shannon complexity captures the morphological changes induced by land-use change rather well. More precisely, the sugarcane field sites lie in the area that has been converted from Atlantic forest to plantation, and the subsequent cultivation activities have brought about changes in the soil morphology. While the results are not entirely consistent in terms of depth and/or position, the 91.7% grouping success rate may be considered quite high, where the discrepancies may be attributed to some yet unknown particularities of these sites.

The approach introduced in the current work does not use arbitrary parameters. It provides a rather precise quantitative complexity measure, which may be seen as a quantifier of soil degradation level. Finally, the novel normalization procedure of variables for representation in the Fisher–Shannon information plane, i.e., preserving the Fisher–Shannon complexity, may be useful for time series and image analysis in general.

## Figures and Tables

**Table 1:** Complexity (distance from the isocomplexity plane).

Sugarcane					
**SC1-10**	**SC2-10**	**SC3-10**	**SC4-10**	**SC5-10**	**SC6-10**
0.0676	0.2899	0.0696	0.0619	0.0825	0.0531
**SC1-20**	**SC2-20**	**SC3-20**	**SC4-20**	**SC5-20**	**SC6-20**
0.0342	0.0951	0.0420	0.1144	0.0714	0.0660
**Atlantic forest**					
**AF1-10**	**AF2-10**	**AF3-10**	**AF4-10**	**AF5-10**	**AF6-10**
0.3290	0.3198	0.2961	0.3022	0.3179	0.1799
**AF1-20**	**AF2-20**	**AF3-20**	**AF4-20**	**AF5-20**	**AF6-20**
0.0998	0.1341	0.1032	0.1704	0.2294	0.1215

## Data Availability

Not applicable.

## Appendix A

Descriptive statistics (minimum, maximum, quartiles, mean, and standard deviation) of the $I_{x}$, $N_{x}$, and $C_{x}$ values of vertical lines.

**Table A1:** Descriptive statistics of Fisher information measure $I_{x}$.

	Min	Q1	Q2	Q3	Max	Mean	Stdev
**Atlantic Forest**							
**AF1-10**	4.24 × 10^−6^	6.07 × 10^−6^	6.50 × 10^−6^	7.01 × 10^−6^	1.62 × 10^−5^	6.62 × 10^−6^	8.67 × 10^−7^
**AF2-10**	3.53 × 10^−6^	6.79 × 10^−6^	7.52 × 10^−6^	8.40 × 10^−6^	1.76 × 10^−5^	7.70 × 10^−6^	1.29 × 10^−6^
**AF3-10**	3.65 × 10^−6^	6.28 × 10^−6^	6.89 × 10^−6^	7.64 × 10^−6^	1.79 × 10^−5^	7.05 × 10^−6^	1.09 × 10^−6^
**AF4-10**	3.31 × 10^−6^	6.54 × 10^−6^	7.14 × 10^−6^	7.89 × 10^−6^	1.69 × 10^−5^	7.34 × 10^−6^	1.19 × 10^−6^
**AF5-10**	4.13 × 10^−6^	7.13 × 10^−6^	7.90 × 10^−6^	8.81 × 10^−6^	2.02 × 10^−5^	8.08 × 10^−6^	1.36 × 10^−6^
**AF6-10**	3.79 × 10^−6^	9.58 × 10^−6^	1.07 × 10^−5^	1.19 × 10^−5^	2.59 × 10^−5^	1.08 × 10^−5^	1.86 × 10^−6^
**AF1-20**	4.51 × 10^−6^	7.46 × 10^−6^	8.14 × 10^−6^	8.86 × 10^−6^	1.46 × 10^−5^	8.20 × 10^−6^	1.03 × 10^−6^
**AF2-20**	3.32 × 10^−6^	8.10 × 10^−6^	9.02 × 10^−6^	1.01 × 10^−5^	1.78 × 10^−5^	9.15 × 10^−6^	1.47 × 10^−6^
**AF3-20**	3.45 × 10^−6^	7.63 × 10^−6^	8.37 × 10^−6^	9.20 × 10^−6^	1.74 × 10^−5^	8.47 × 10^−6^	1.19 × 10^−6^
**AF4-20**	3.93 × 10^−6^	7.47 × 10^−6^	8.20 × 10^−6^	9.04 × 10^−6^	1.58 × 10^−5^	8.31 × 10^−6^	1.18 × 10^−6^
**AF5-20**	3.69 × 10^−6^	7.33 × 10^−6^	8.18 × 10^−6^	9.21 × 10^−6^	1.82 × 10^−5^	8.37 × 10^−6^	1.50 × 10^−6^
**AF6-20**	4.08 × 10^−6^	8.11 × 10^−6^	9.08 × 10^−6^	1.02 × 10^−5^	1.82 × 10^−5^	9.22 × 10^−6^	1.54 × 10^−6^
**Sugarcane**							
**SC1-10**	4.91 × 10^−6^	8.15 × 10^−6^	8.76 × 10^−6^	9.40 × 10^−6^	1.45 × 10^−5^	8.80 × 10^−6^	9.37 × 10^−7^
**SC2-10**	3.24 × 10^−6^	8.35 × 10^−6^	9.35 × 10^−6^	1.05 × 10^−5^	2.02 × 10^−5^	9.47 × 10^−6^	1.60 × 10^−6^
**SC3-10**	3.57 × 10^−6^	9.05 × 10^−6^	9.78 × 10^−6^	1.05 × 10^−5^	1.61 × 10^−5^	9.81 × 10^−6^	1.13 × 10^−6^
**SC4-10**	4.52 × 10^−6^	9.02 × 10^−6^	9.77 × 10^−6^	1.06 × 10^−5^	1.71 × 10^−5^	9.83 × 10^−6^	1.16 × 10^−6^
**SC5-10**	4.01 × 10^−6^	9.22 × 10^−6^	1.01 × 10^−5^	1.10 × 10^−5^	1.82 × 10^−5^	1.01 × 10^−5^	1.31 × 10^−6^
**SC6-10**	3.67 × 10^−6^	1.07 × 10^−5^	1.17 × 10^−5^	1.27 × 10^−5^	1.94 × 10^−5^	1.17 × 10^−5^	1.45 × 10^−6^
**SC1-20**	5.11 × 10^−6^	1.03 × 10^−5^	1.11 × 10^−5^	1.19 × 10^−5^	1.72 × 10^−5^	1.11 × 10^−5^	1.15 × 10^−6^
**SC2-20**	3.51 × 10^−6^	8.54 × 10^−6^	9.36 × 10^−6^	1.02 × 10^−5^	1.81 × 10^−5^	9.43 × 10^−6^	1.30 × 10^−6^
**SC3-20**	3.46 × 10^−6^	9.87 × 10^−6^	1.07 × 10^−5^	1.15 × 10^−5^	1.78 × 10^−5^	1.07 × 10^−5^	1.24 × 10^−6^
**SC4-20**	3.96 × 10^−6^	7.93 × 10^−6^	8.71 × 10^−6^	9.57 × 10^−6^	1.72 × 10^−5^	8.81 × 10^−6^	1.26 × 10^−6^
**SC5-20**	3.71 × 10^−6^	9.17 × 10^−6^	1.01 × 10^−5^	1.11 × 10^−5^	1.79 × 10^−5^	1.01 × 10^−5^	1.43 × 10^−6^
**SC6-20**	6.56 × 10^−6^	1.27 × 10^−5^	1.41 × 10^−5^	1.56 × 10^−5^	2.68 × 10^−5^	1.42 × 10^−5^	2.17 × 10^−6^

**Table A2:** Descriptive statistics of Shannon entropy power $N_{x}$.

	Min	Q1	Q2	Q3	Max	Mean	Stdev
**Atlantic Forest**							
**AF1-10**	1.24 × 10^5^	2.14 × 10^5^	2.37 × 10^5^	2.62 × 10^5^	4.10 × 10^5^	2.39 × 10^5^	3.53 × 10^4^
**AF2-10**	7.07 × 10^4^	1.84 × 10^5^	2.11 × 10^5^	2.39 × 10^5^	6.14 × 10^5^	2.13 × 10^5^	4.03 × 10^4^
**AF3-10**	7.43 × 10^4^	1.92 × 10^5^	2.17 × 10^5^	2.44 × 10^5^	6.00 × 10^5^	2.20 × 10^5^	3.99 × 10^4^
**AF4-10**	9.73 × 10^4^	1.85 × 10^5^	2.09 × 10^5^	2.37 × 10^5^	6.23 × 10^5^	2.15 × 10^5^	4.63 × 10^4^
**AF5-10**	8.54 × 10^4^	1.76 × 10^5^	2.03 × 10^5^	2.34 × 10^5^	3.88 × 10^5^	2.06 × 10^5^	4.05 × 10^4^
**AF6-10**	5.55 × 10^4^	1.04 × 10^5^	1.30 × 10^5^	1.65 × 10^5^	4.34 × 10^5^	1.38 × 10^5^	4.11 × 10^4^
**AF1-20**	7.64 × 10^4^	1.30 × 10^5^	1.45 × 10^5^	1.62 × 10^5^	3.72 × 10^5^	1.48 × 10^5^	2.40 × 10^4^
**AF2-20**	6.30 × 10^4^	1.22 × 10^5^	1.39 × 10^5^	1.60 × 10^5^	7.27 × 10^5^	1.43 × 10^5^	3.08 × 10^4^
**AF3-20**	5.92 × 10^4^	1.25 × 10^5^	1.41 × 10^5^	1.58 × 10^5^	9.73 × 10^5^	1.44 × 10^5^	2.84 × 10^4^
**AF4-20**	7.90 × 10^4^	1.40 × 10^5^	1.58 × 10^5^	1.82 × 10^5^	3.96 × 10^5^	1.64 × 10^5^	3.44 × 10^4^
**AF5-20**	6.98 × 10^4^	1.47 × 10^5^	1.72 × 10^5^	2.03 × 10^5^	5.56 × 10^5^	1.78 × 10^5^	4.29 × 10^4^
**AF6-20**	5.91 × 10^4^	1.18 × 10^5^	1.36 × 10^5^	1.58 × 10^5^	5.51 × 10^5^	1.39 × 10^5^	2.98 × 10^4^
**Sugarcane**							
**SC1-10**	7.31 × 10^4^	1.19 × 10^5^	1.29 × 10^5^	1.41 × 10^5^	3.03 × 10^5^	1.31 × 10^5^	1.71 × 10^4^
**SC2-10**	6.34 × 10^4^	1.40 × 10^5^	1.72 × 10^5^	2.13 × 10^5^	9.73 × 10^5^	1.79 × 10^5^	5.13 × 10^4^
**SC3-10**	6.82 × 10^4^	1.06 × 10^5^	1.16 × 10^5^	1.29 × 10^5^	8.74 × 10^5^	1.20 × 10^5^	2.02 × 10^4^
**SC4-10**	6.50 × 10^4^	1.05 × 10^5^	1.15 × 10^5^	1.28 × 10^5^	4.23 × 10^5^	1.17 × 10^5^	1.82 × 10^4^
**SC5-10**	6.23 × 10^4^	1.02 × 10^5^	1.14 × 10^5^	1.31 × 10^5^	4.34 × 10^5^	1.20 × 10^5^	2.60 × 10^4^
**SC6-10**	5.43 × 10^4^	8.70 × 10^4^	9.63 × 10^4^	1.08 × 10^5^	7.01 × 10^5^	9.86 × 10^4^	1.63 × 10^4^
**SC1-20**	6.05 × 10^4^	9.01 × 10^4^	9.72 × 10^4^	1.05 × 10^5^	3.58 × 10^5^	9.84 × 10^4^	1.22 × 10^4^
**SC2-20**	5.79 × 10^4^	1.12 × 10^5^	1.26 × 10^5^	1.43 × 10^5^	1.05 × 10^6^	1.30 × 10^5^	2.68 × 10^4^
**SC3-20**	5.88 × 10^4^	9.36 × 10^4^	1.02 × 10^5^	1.12 × 10^5^	1.10 × 10^6^	1.04 × 10^5^	1.54 × 10^4^
**SC4-20**	6.46 × 10^4^	1.24 × 10^5^	1.39 × 10^5^	1.57 × 10^5^	4.46 × 10^5^	1.43 × 10^5^	2.69 × 10^4^
**SC5-20**	5.79 × 10^4^	1.00 × 10^5^	1.12 × 10^5^	1.27 × 10^5^	5.15 × 10^5^	1.17 × 10^5^	2.52 × 10^4^
**SC6-20**	3.83 × 10^4^	7.48 × 10^4^	8.49 × 10^4^	9.66 × 10^4^	2.19 × 10^5^	8.69 × 10^4^	1.72 × 10^4^

**Table A3:** Descriptive statistics of Fisher–Shannon complexity $C_{x}$.

	Min	Q1	Q2	Q3	Max	Mean	Stdev
**Atlantic Forest**							
**AF1-10**	1.05	1.36	1.51	1.72	4.30	1.58	3.22 × 10^−1^
**AF2-10**	1.02	1.36	1.53	1.79	4.61	1.63	3.89 × 10^−1^
**AF3-10**	1.03	1.33	1.46	1.65	4.13	1.54	3.11 × 10^−1^
**AF4-10**	1.03	1.30	1.44	1.68	5.08	1.58	4.53 × 10^−1^
**AF5-10**	1.02	1.35	1.54	1.84	4.69	1.65	4.22 × 10^−1^
**AF6-10**	1.01	1.14	1.32	1.64	5.48	1.47	4.74 × 10^−1^
**AF1-20**	1.01	1.12	1.17	1.24	2.29	1.19	1.00 × 10^−1^
**AF2-20**	1.01	1.14	1.21	1.33	4.13	1.29	2.69 × 10^−1^
**AF3-20**	1.01	1.11	1.16	1.23	4.13	1.20	1.61 × 10^−1^
**AF4-20**	1.01	1.18	1.27	1.40	3.58	1.34	2.55 × 10^−1^
**AF5-20**	1.01	1.22	1.34	1.55	4.85	1.47	4.17 × 10^−1^
**AF6-20**	1.01	1.13	1.21	1.31	2.71	1.25	1.68 × 10^−1^
**Sugarcane**							
**SC1-10**	1.01	1.09	1.12	1.17	2.00	1.14	7.35 × 10^−2^
**SC2-10**	1.01	1.29	1.50	1.91	4.44	1.68	5.32 × 10^−1^
**SC3-10**	1.01	1.08	1.12	1.20	3.55	1.16	1.19 × 10^−1^
**SC4-10**	1.01	1.08	1.11	1.17	2.38	1.14	9.14 × 10^−2^
**SC5-10**	1.01	1.08	1.12	1.21	3.79	1.19	2.29 × 10^−1^
**SC6-10**	1.00	1.07	1.11	1.17	2.87	1.14	1.02 × 10^−1^
**SC1-20**	1.00	1.05	1.07	1.10	1.97	1.08	5.51 × 10^−2^
**SC2-20**	1.01	1.10	1.16	1.25	4.03	1.20	1.43 × 10^−1^
**SC3-20**	1.01	1.05	1.08	1.12	4.82	1.10	6.40 × 10^−2^
**SC4-20**	1.01	1.14	1.20	1.28	3.19	1.23	1.34 × 10^−1^
**SC5-20**	1.01	1.08	1.12	1.19	3.36	1.15	1.27 × 10^−1^
**SC6-20**	1.00	1.11	1.18	1.27	2.73	1.20	1.28 × 10^−1^
